# Estimation of losses of quality-adjusted life expectancy attributed to the combination of cognitive impairment and multimorbidity among Chinese adults aged 45 years and older

**DOI:** 10.1186/s12889-020-10069-w

**Published:** 2021-01-05

**Authors:** Suting Xiong, Siyuan Liu, Yanan Qiao, Dingliu He, Chaofu Ke, Yueping Shen

**Affiliations:** Department of Epidemiology and Biostatistics, School of Public Health, Medical College of Soochow University, 199 Renai Road, Suzhou, 215123 People’s Republic of China

**Keywords:** Quality-adjusted life expectancy, Cognitive impairment, Multimorbidity, CHARLS

## Abstract

**Objectives:**

This study aims to estimate the losses of quality-adjusted life expectancy (QALE) due to the joint effects of cognitive impairment and multimorbidity, and to further confirm additional losses attributable to this interaction among middle-aged and elderly Chinese people.

**Methods:**

The National Cause of Death Monitoring Data were linked with the China Health and Retirement Longitudinal Study (CHARLS). A mapping and assignment method was used to estimate health utility values, which were further used to calculate QALE. Losses of QALE were measured by comparing the differences between subgroups. All the losses of QALE were displayed at two levels: the individual and population levels.

**Results:**

At age 45, the individual-level and population-level losses of QALE attributed to the combination of cognitive impairment and multimorbidity were 7.61 (95% CI: 5.68, 9.57) years and 4.30 (95% CI: 3.43, 5.20) years, respectively. The losses for cognitive impairment alone were 3.10 (95% CI: 2.29, 3.95) years and 1.71 (95% CI: 1.32, 2.13) years at the two levels. Similarly, the losses for multimorbidity alone were 3.53 (95% CI: 2.53, 4.56) years and 1.91 (95% CI: 1.24, 2.63) years at the two levels. Additional losses due to the interaction of cognitive impairment and multimorbidity were indicated by the 0.98 years of the individual-level gap and 0.67 years of the population-level gap.

**Conclusion:**

Among middle-aged and elderly Chinese people, cognitive impairment and multimorbidity resulted in substantial losses of QALE, and additional QALE losses were seen due to their interaction at both individual and population levels.

## Background

Age-associated cognitive impairment is a transitional link between healthy ageing and dementia, featuring declines in memory, attention, and cognitive function, with a 10% conversion rate from impaired status to the diagnosis of dementia [1]. At the end of 2019, the population aged 65 and above in China accounted for 176.0 million [2]. A large­sample, multi-region study showed that the prevalence of total dementia in the population aged 65 years and older in China was approximately 5 % in 2019 [3]. Moreover, mild cognitive impairment (MCI) is estimated to be > 4 times more common than dementia [1, 4].

Meanwhile, age-dependent noncommunicable diseases (NCD) have been proved to experience a continuous increase among elderly individuals in recent decades [5]. Nearly 50% of the NCD burden in China occurs in people aged 65 years and older [6], while 81.3% [7] of this group of older adults have ≥2 chronic conditions [8]. Multimorbidity, which is defined as the co-occurrence of two or more chronic diseases in an individual, is widely observed beyond two-thirds of older adults [9, 10]. It is known that individuals with memory-related problems (e.g. Alzheimer’s disease, brain atrophy, Parkinson’s disease) usually have cognitive impairment to different degrees. In this work, multimorbidity was defined as the total number of self-reported chronic conditions we could collect, except for memory-related problems to avoid overlapping estimates. Many clinical studies have suggested that older adults with at least two diseases are more susceptible to developing cognitive impairment, than those without multimorbidity [11–15]. The hypothesis that multimorbidity may increase the risk of cognitive impairment has also been verified in population-based studies [16–19].

However, few studies have been conducted to measure how long elderly individuals would be expected to live less in a quality damaged state caused by this combined burden. Quality-adjusted life expectancy (QALE), as a more sensitive and comprehensive population-health measure, combines health-related quality of life (HRQoL) with life expectancy (LE) to obtain a single summary score [20–22]. When it is difficult to estimate HRQoL directly, mapping methods could help generate predictive utility values based on existing health-related data [23–25]. Some researchers argue that QALE is better for public health surveillance among older adults than other health expectancy measures [21, 23]. Previous studies [26, 27] have demonstrated that QALE losses can quantify the difference in disease burden due to cause-specific mortality and morbidity, and could be displayed at both the individual and population levels [26]. Similar to the definition of attributable risk (AR) and population attributable risk (PAR) in epidemiology [28, 29], losses of QALE could be measured at both individual and population levels [26]. For instance, the definition of individual-level losses of QALE due to cognitive impairment is referred to as the difference in QALE between groups with and without cognitive impairment. The population-level losses of QALE are considered the difference in QALE between the group with cognitive impairment and the total population. Losses of QALE due to the combination of cognitive impairment and multimorbidity could be estimated in the same way.

The Chinese population is ageing dramatically. The proportion of people aged 45 to 64 years in China’s total population was approximately 36.8% in 2018 [30], and the prevalence of multimorbidity was at least 51.6% for middle-aged adults (45 to 59 years old) [7]. Moreover, studies have also shown that cognitive problems are common in the middle-aged population in China [31–34]. Therefore, it is of great significance to evaluate QALE due to multimorbidity and cognitive impairment in the middle-aged population in China.

Based on the availability of the China Health and Retirement Longitudinal Study (CHARLS) data (including 17,707 adults aged 45 years and older), this study aims to 1) estimate the losses of QALE attributed to the combination of cognitive impairment and multimorbidity at both the individual and population levels; and 2) confirm the additional losses of QALE due to the interaction of cognitive impairment and multimorbidity. Studies about losses of QALE quantifying the severity of health damage are valuable for both clinical intervention assessments over a predetermined time interval and resource optimization in public health strategies for those in high-risk groups [23, 35].

## Methods

A total of 17,224 individuals aged 45 years and older (mean age: 59 years and standard deviation: 9.9 years) at the baseline wave (2011) of CHARLS were used to estimate the HRQoL among the included participants. The follow-up data of these participants (2013, 2015) were used to estimate the cause-specific mortality rates of cognitive impairment and multimorbidity [36]. Brief, these three-wave surveys of sampling residents aged 45 years to 115 years in China were conducted through face-to-face computer-assisted personal interviewing in biennial summer. The detailed profile [37] and published CHARLS data are available in the CHARLS repository, http://charls.pku.edu.cn/index/zh-cn.html.

### Measurement of cognitive impairment

To assess cognitive impairment, we used the two-part brief cognition measurement sets of the CHARLS [38–40], similar to the imputed cognition part of the American Health and Retirement Study (HRS). The first part evaluates episodic memory through a calculation of average scores (0–10) among 10 Chinese words of immediate and delayed recall. The second part measures executive function based on an 11-score sum, which consists of the orientation of dates (day, week, month, season, and year), serial subtraction of 7 from 100 five times successively, and an item of repainting a specific picture. The current study evaluated the cognitive function of the participants by calculating the total score of both parts, which ranged from 0 to 21 [38, 41, 42]. The diagnosis of memory-related problems was investigated face-to-face by the “doctor diagnosed health problems” part, which was also included in the CHARLS. Therefore, the cut-off value for the judgement of cognitive impairment was estimated by a receiver operating characteristic (ROC) curve analysis combining the cognitive scores (0–21) and the diagnosis of memory-related problems.

### Definition of multimorbidity

The doctor diagnosed health problems part of the CHARLS covered 14 chronic conditions diagnosed by doctors: hypertension, diabetes or high blood sugar, cancer or a malignant tumour, chronic lung disease, stroke, other cardiovascular problems, emotional or psychiatric problems, arthritis, dyslipidaemia, liver diseases, kidney diseases, digestive diseases, asthma, and memory-related diseases [37]. More detailed definitions of these 14 conditions can be found in the data using documents provided on their website [43]. According to the most common approach [41], this study defined multimorbidity as a count of the number of diseases without weighting for severity [44]. As mentioned above, the memory-related problem was removed from the 14 types of chronic conditions.

### Health utility value – morbidity rate

To describe the HRQoL using a summary value between 0 (for death) and 1 (for perfect health), a preference-based measure – the health utility value – was used to estimate the impacts of physical and mental dysfunction [45]. This study obtained health utility values by a nonparametric mapping method.

From a total of 17,224 individuals (aged ≥45 years) included in CHARLS, 3636 random participants answered five health profile questions at the baseline wave (2011), which were analogous to the five domains of the EuroQol-5 Dimensions (EQ-5D) instrument. The descriptive system of the EQ-5D classifies people’s health into 1 of 5 levels in 5 domains: anxiety/depression, pain/discomfort, usual activities, self-care, and mobility [46]. We constructed the EQ-5D-5L scale based on similar variables covered in the baseline data of CHARLS. Then, we obtained each corresponding utility value through an EQ-5D-5L utility database (a full set of predicted values for all 3125 health states) for China [47]. Cronbach’s alpha and confirmatory factor analyses were performed to test the reliability and validity of the constructed scale.

Next, a propensity score matching (PSM) based mapping method was used to assign health utility values to matched participants, who had no health utility values [48]. From 17,224 individuals aged ≥45 years, 3600 participants had complete data on covariables and health utility values, and 11,850 participants had complete information on covariables of propensity score matching (PSM) without health utility values. The covariables used for the PSM were demographic characteristics (including 5-year-interval age groups, gender, marital status, educational level, and residence status) and other health-related quality of life (HRQoL) items (including the scores of the 6-item ADL scale, 5-item IADL scale, 7-item mobility scale, 10-item CESD scale, 5-item chest pain scale, and 5-item SroH scale) in the CHARLS. Under the control of the 1:3 matching ratio and the 0.01 calliper value, 10,214 out of 11,850 participants were assigned health utility values. The balance of the PSM-based assignment method was examined by multiple logistic regression.

Of 13,850 individuals with health utility values, 12,300 with complete information on cognition were used to estimate the average health utility values in age-specific intervals (9 five-year intervals), replacing morbidity. The bootstrapping-based estimates of confidence intervals for the average health utility values were computed from the 2.5th to the 97.5th percentiles, and confidence intervals were computed for the differences in the average health utility values (2.5th, 97.5th).

### Cause-specific mortality rate

The age-specific death rate (m) was derived from the national cause of death monitoring data (2011) [49]. However, age-specific death rates stratified by cognitive impairment and multimorbidity were not available, so these rates were estimated through the following formulas. For example, death rates for those with cognitive impairment (m_1_) and those without cognitive impairment (m_0_) were calculated using the hazard ratio (h) of dying for cognitive impairment versus no cognitive impairment and the prevalence of cognitive impairment (p) by ${m}_1=\frac{hm}{hp+\left(1-p\right)}$ and ${m}_0=\frac{m}{hp+\left(1-p\right)}$, respectively [26]. Likewise, the death rates for the combination of cognitive impairment and multimorbidity were estimated through the same formulas listed above. Based on the Cox proportional hazards model, hazard ratios were computed. The prevalence of cognitive impairment and multimorbidity obtained from the CHARLS data were only assessed starting at age 45.

### QALE and losses of QALE

**Fig. 1:**
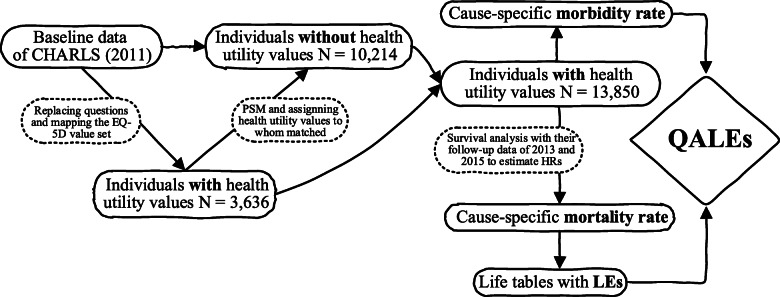
Diagram for the calculation of QALE. CHARLS (2011), baseline data of the China Health and Retirement Longitudinal Study; PSM, propensity score matching; HR, hazard ratio; LE, life expectancy; QALE, quality-adjusted life expectancy

The life table of the general population was constructed with the age-specific mortality rates from the national cause of death monitoring data (2011) [49]. Based on the cause-specific mortality rate, the life tables of the subgroups were constructed. Let *A*_*i*_ be the number of the population surviving to age *i* (*i* ≥ 45). The quality-adjusted life-years (QALYs) *D*_*i*_*y*_*i*_ in the age-specific interval [*i, i* + 5] were calculated using the average health utility value *y*_*i*_ and the person-year survival *D*_*i*_ in the age-specific interval [*i, i* + 5] so that QALE_*i*_ at age *x* was calculated by ${\mathrm{QALE}}_i=\sum \limits_{i\ge x}{D}_i{y}_i/{A}_i\kern1em i,x\in \left[45,80\right]$ [22, 26]. An entire process regarding the estimation of QALE is presented in Fig. 1. All the losses of QALE were displayed at two levels: the individual and the population level. Through the confidence intervals of health utility values and their differences, the confidence intervals for QALE and losses of QALE were computed.

### Sensitivity analysis

Two sensitivity analyses were conducted to evaluate the impact of missing values. Different from the above analysis that excluded participants (*n* = 1550) without cognitive scores, we classified all participants missing cognitive information into either the low cognition level or high cognition level for two sensitivity analyses. According to the new grouping of cognitive impairment, we estimated the QALE losses.

## Results

### Characteristics of participants

According to the ROC curve results, the optimal cut-off value for judging cognitive impairment was 8.25, and the AUC for this value was 0.613 (95% CI: 0.575, 0.650). All participants (*n* = 13,850) in this study were divided into four subgroups with another missing cognitive subgroup by the combination of cognitive impairment and multimorbidity. Characteristics in these subgroups are presented in Appendix Table 1. Participants featuring higher age, female sex, divorced/separated status, lower education level, living in urban areas, smoking, drinking, lower BMI, and multimorbidity, were more likely to have low-level-cognition. However, the characteristics described in the missing cognitive subgroup were similar to the subgroups of low-level-cognition.

### Results of mapping and assigning values

Based on the results of mapping, the Cronbach’s alpha based on standardized items (ɑ’ = 0.829) and the results of the confirmatory factor analysis (with five eigenvalues obliquely rotated ≥1 corresponding to the five dimensions of the EQ-5D) reflected excellent reliability and validity. The results of assigning values based on PSM were examined by multiple logistic regression, showing a good balance in almost all of the covariates of PSM between participants with health utility values and those who were assigned health utility values after PSM matching (Table 1). Except for married or partnered status which was more likely to be matched (*P* = 0.028), other covariates of PSM had no statistical significance between the two groups (*P* > 0.05), particularly the differences in health utility values with no significance (*P* = 0.124).

**Table 1:** Results of balance between two groups of participants after PSM-based assigning values by multiple logistic regression

Covariates of PSM	Participants with complete information on multimorbidity and cognitive function (*n* = 12,300)				
	Participants owned utility values		Participants were given utility values after PSM matching		*P* value (ɑ = 0.05)
	(*n* = 3211) (%)^a^		(*n* = 9089) (%)^a^		
Age groups (y), n (%)					0.609
45–64	2390	(74.43)	6850	(74.43)	
65–84	743	(22.20)	2018	(23.14)	
≥ 80	78	(2.43)	221	(2.43)	
Women, n (%)	1700	(52.94)	4722	(51.95)	0.526
Married & partnered, n (%)	2791	(86.92)	8058	(88.66)	**0.028**
Education, n (%)					0.567
Less than lower secondary	2775	(86.42)	7893	(86.84)	
Upper secondary & vocational training	359	(11.18)	972	(10.69)	
tertiary	76	(2.37)	224	(2.47)	
Missing	1	(0.03)	0	(0.00)	
Residence, n (%)					0.892
Rural Village	2446	(76.18)	6950	(76.47)	
Urban Community	762	(23.73)	2139	(23.53)	
Missing	3	(0.09)	0	(0.00)	
ADL-6 item Scale, mean (SD)	0.28	(0.82)	0.27	(0.81)	0.284
IADL-5 item Scale, mean (SD)	0.38	(0.93)	0.38	(0.93)	0.650
Mobility-7 item Scale, mean (SD)	1.15	(1.44)	1.16	(1.44)	0.499
CESD-30 item Scale, mean (SD)	8.34	(6.26)	8.22	(6.31)	0.244
5-item of Chestpain, mean (SD)	1.53	(1.05)	1.52	(1.04)	0.671
5-item of SroH, mean (SD)	3.56	(0.92)	3.58	(0.93)	0.436
health utility values of EQ-5D, mean (SD)	0.87	(0.19)	0.86	(0.20)	0.124

*PSM* propensity score matching;

*ADL* activities of daily living, *IADL* instrumental activities of daily living, *CESD* Centre for Epidemiology Studies-Depression Scale;

*SroH* self-report of health, *SD* standard deviation;

^a^ Percentages accounting for sampling proportion in subgroups

### Quality-adjusted life expectancy (QALE)

From age of 45 to 85 years, the QALE decreased by the age intervals in the four subgroups. However, the declining rate in the low-level-cognition with multimorbidity subgroup was the fastest of the four subgroups (Table 2). A QALE of more than 20 years was only seen with the first two age intervals (45–49 and 50–54) in the first subgroup, in contrast to other subgroups, which had QALE > 20 years in at least at three age intervals. At the age interval of 70–74 years, the QALE in the other three subgroups was > 10 years, but people with multimorbidity and cognitive impairment had an 8.86-year QALE at the same age interval. At > 85 years of age, the QALE in participants with multimorbidity and cognitive impairment was 1/3 less than those without multimorbidity and cognitive impairment (2.01 years vs. 6.24 years). The QALE results of all age intervals are described in Table 2.

**Table 2:** QALE among different subgroups divided by the combination of cognitive impairment and multimorbidity

Age intervals (y)	QALEs among the different subgroups (*n* = 13,850)				
	With multimorbidity (*n* = 6087)		Without multimorbidity (*n* = 6213)		Missing value (*n* = 1550) (95% CI)
	Low cognition (*n* = 1766) (95% CI)	High cognition (*n* = 4321) (95% CI)	Low cognition (*n* = 1490) (95% CI)	High cognition (*n* = 4723) (95% CI)	
45–49	25.73 (25.27, 26.18)	29.52 (29.08, 29.95)	29.62 (29.17, 30.04)	33.34 (32.93, 33.74)	28.12 (27.63, 28.59)
50–54	22.11 (21.70, 22.50)	25.61 (25.23, 25.99)	25.55 (25.15, 25.93)	29.25 (28.88, 29.62)	24.09 (23.64, 24.53)
55–59	18.36 (18.01, 18.69)	21.73 (21.39, 22.06)	21.49 (21.15, 21.83)	25.10 (24.78, 25.43)	20.09 (19.69, 20.48)
60–64	14.87 (14.58, 15.14)	17.96 (17.67, 18.24)	17.70 (17.41, 17.98)	21.24 (20.96, 21.52)	16.59 16.26, 16.92)
65–69	11.84 (11.61, 12.06)	14.60 (14.36, 14.84)	14.17 (13.93, 14.38)	17.68 (17.44, 17.91)	13.17 (12.88, 13.45)
70–74	8.86 (8.67, 9.03)	11.48 (11.29, 11.67)	11.02 (10.82, 11.19)	14.37 (14.19, 14.55)	10.00 (9.77, 10.22)
75–79	6.06 (5.92, 6.19)	8.46 (8.31, 8.61)	8.35 (8.20, 8.49)	11.46 (11.33, 11.58)	7.17 (7.00, 7.33)
80–84	3.70 (3.61, 3.79)	5.97 (5.87, 6.07)	5.85 (5.73, 5.96)	8.79 (8.69, 8.88)	4.63 (4.51, 4.74)
85+	2.01 (1.96, 2.05)	4.19 (4.16, 4.23)	3.59 (3.49, 3.66)	6.24 (6.19, 6.30)	2.55 (2.50, 2.60)

*CI* confidence interval;

*QALE* quality-adjusted life expectancy

### Losses of QALE

**Fig. 2:**
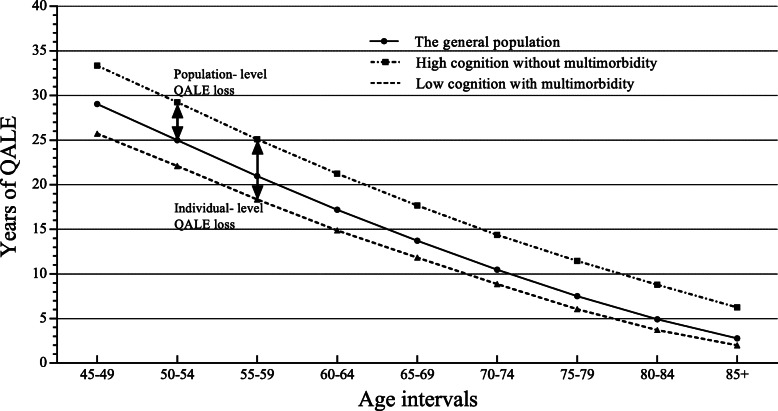
QALE tendency among different groups. Population-level QALE loss: the difference in QALE between the population with the combination of cognitive impairment and multimorbidity and the general population; individual-level QALE loss: the difference in QALE between the population with the combination of cognitive impairment and multimorbidity and the population without this combination

The differences in the three trend lines among these nine age intervals showed QALE losses at both the individual and population levels due to the joint effect of cognitive impairment and multimorbidity (Fig. 2). From 45 to 85 years of age, the individual-level QALE losses derived from the combined burden of cognitive impairment and multimorbidity were approximately 7.6 to 4.2 years, and the corresponding population-level QALE losses were 4.3 to 3.5 years. The QALE losses due to multimorbidity alone were consistently larger than the losses due to cognitive impairment in all age intervals at both levels.

**Fig. 3:**
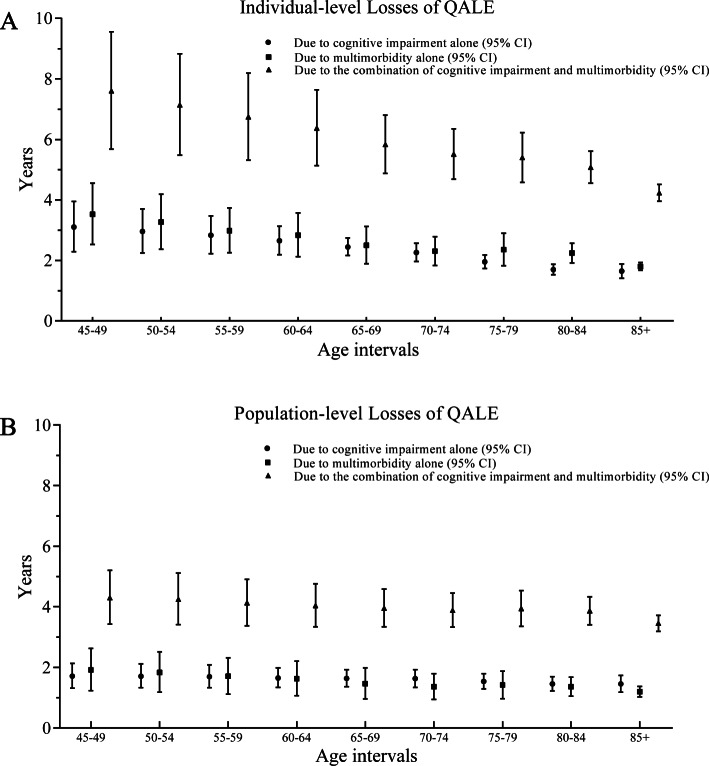
Losses of QALE (with corresponding 95% confidence intervals). These losses are displayed for cognitive impairment, multimorbidity and the combination of cognitive impairment and multimorbidity at the individual level (3A) and population level (3B)

When comparing the high-level cognition without multimorbidity group with the low-level-cognition with multimorbidity group, the individual-level QALE loss (age 45 years) was 7.61 (95% CI: 5.68, 9.57) years. Analogously, the QALE loss for cognitive impairment alone was 3.10 (95% CI: 2.29, 3.95) years, and the QALE loss for multimorbidity alone was 3.53 (95% CI: 2.53, 4.56) years. According to Fig. 3b, the population-level loss of QALE derived from the two groups (the high-level cognition without multimorbidity group and the general population group) was 4.30 (95% CI: 3.43, 5.20) years. At the same age interval, compared with the general population group, the QALE loss for cognitive impairment alone was 1.71 (95% CI: 1.32, 2.13) years, and the QALE loss for multimorbidity alone was 1.91 (95% CI: 1.24, 2.63) years.

Obviously, the 0.98 (= 7.61–3.10-3.53) years for the individual-level gap showed that there were additional losses of QALE due to the interaction of cognitive impairment and multimorbidity; this is the same with the 0.68 (= 4.30–1.71-1.91) years of loss at the population-level. Other results at both levels, which were described in detail for all age intervals, are shown in Appendix Tables 2 and 3.

### Results of the sensitivity analysis

When classifying all participants without the cognitive information into the low cognition level, there was no evident impact on the results at the individual (Appendix Table 4) or population (Appendix Table 5) levels. Similar results were seen at the individual (Appendix Table 6) and population (Appendix Table 7) levels in another sensitivity analysis (classifying all participants without the cognitive information into the high cognition level).

## Discussion

The current study measured the losses of QALE due to the combination of cognitive impairment and multimorbidity, and then discovered a significantly additional burden from the interaction of cognitive impairment and multimorbidity at both the individual and population levels.

Disease-specific QALE has previously been evaluated in several clinical studies [27, 50–53] and population-based studies [54–57]. In a study by Jia et al. [57], the authors found that the individual-level QALE losses derived from diabetes, hypertension, asthma, heart disease, and stroke were 8.9 years, 4.3 years, 6.4 years, 7.9 years, and 9.2 years, respectively, at the same age interval in America. However, the losses of QALE attributed to multimorbidity have not been assessed thus far. Moreover, the HRQoL for cognitively impaired individuals has been measured in several studies [58–64]. Nevertheless, there have been no studies focusing on cognitive impairment measured by QALE. Likewise, we could not find studies to date focusing on the measurement of QALE losses for the combination of multimorbidity and cognitive impairment.

The declining rates of QALE by age were different among the four subgroups, which suggested the different health burden caused by different exposure conditions and age stages. Obviously, people with both cognitive impairment and multimorbidity had the lowest QALE and presented the fastest rate of decline for the QALE. Moreover, it seemed that the individual-level losses of QALE paradoxically declined as age increased (Appendix Table 2, Fig. 3a). The underlying mechanism could be explained by “the compression of morbidity” [65]. The competitive nature of mortality in the calculation means that higher mortality could cause a lower QALE. When health damage contributes to both mortality and morbidity, individuals with an inferior health status (e.g., the cognitive impairment and multimorbidity subgroup) and higher age intervals could be more likely to die than to live. Population-level losses reflect the difference between the unexposed group and the whole population, which could neutralize the stronger competitive effect of mortality in higher age intervals to some extent. Actually, the losses at the population-level remained comparatively steady (Appendix Table 3, Fig. 3b), which confirmed that the health damage due to the combination of cognitive impairment and multimorbidity could not be weakened by age. However, there were still large QALE losses in the middle-aged groups, which suggests that early preventive measures for people aged 45 years and older may be worthwhile.

Several studies have explored the biological mechanisms for the acceleration of dementia progression by multimorbidity [16, 66–69]. These studies found that amyloid aggregation, vascular damage, drug-disease interactions, chronic hypoxemia, and peripheral insulin resistance might contribute to this correlation. In addition, care-related and psychosocial factors also operate as determinants of the interaction between cognitive function and multimorbidity [15]. The coexistence of multimorbidity and cognitive impairment makes it particularly challenging for these patients to sufficiently express discomfort/pain. Doctors’ fragmented views of health problems lead to untreated or even undiagnosed chronic conditions in people with dementia [70]. This vicious cycle could result in the suboptimal use of health services and reduced quality of life and survival [71].

This study is the first to calculate QALE and QALE losses as indices for evaluating the burden of cognitive impairment and multimorbidity at different stages of life. Such strategies could allow the direct comparison of the health burden of different diseases, demographic characteristics, risk factors, therapeutic schemes, and health intervention policies [57, 72]. The main limitation refers to the definition of cognitive impairment and multimorbidity. Instead of using the definition of MCI [73], an ROC curve analysis was performed to generate the cut-off score for cognitive impairment in this study, which could underestimate cognitive impairment because of the conservative calculation of the cut-off value. Similarly, the severity of the disease was not adjusted in the “multimorbidity” definition, which was only measured through the number of diseases. Moreover, self-reported chronic conditions may underestimate the prevalence of multimorbidity.

## Conclusion

In conclusion, the current study demonstrated losses of QALE due to the joint effect of cognitive impairment and multimorbidity, and confirmed an additional burden from the interaction of cognitive impairment and multimorbidity at both the individual and population levels. Therefore, this study indicated that more focus and early interventions should be placed on the group with risks of both cognitive impairment and multimorbidity, and these measures should be taken not only for clinical individuals under treatment but also among these high-risk groups in the community.

## Supplementary Information

**Additional file 1: Appendix Table 1.** Characteristics among groups of Chinese people ≥45 years of age included in this study - CHARLS (2011).

**Additional file 2: Appendix Table 2.** Losses of QALE at the individual level (with the corresponding 95% confidence intervals).

**Additional file 3: Appendix Table 3.** Losses of QALE at the population level (with the corresponding 95% confidence intervals).

**Additional file 4: Appendix Table 4.** Losses of QALE (all participants without cognitive information were classified into the low cognition level) at the individual level based on the sensitivity analysis (with the corresponding 95% confidence intervals).

**Additional file 5: Appendix Table 5.** Losses of QALE (all participants without cognitive information were classified into the low cognition level) at the population level based on the sensitivity analysis (with the corresponding 95% confidence intervals).

**Additional file 6: Appendix Table 6.** Losses of QALE (all participants without cognitive information were classified into the high cognition level) at the individual level based on the sensitivity analysis (with the corresponding 95% confidence intervals).

**Additional file 7: Appendix Table 7.** Losses of QALE (all participants without cognitive information were classified into the high cognition level) at the population level based on the sensitivity analysis (with the corresponding 95% confidence intervals).

## Data Availability

The demographic data that support the findings of this study are available from the Chinese National Bureau of Statistics. The other data that support the findings of this study are available in the CHARLS repository (http://charls.pku.edu.cn/en).

## Abbreviations

QALE: Quality-adjusted life expectancy
CHARLS: China Health and Retirement Longitudinal Study
CI: Confidence interval
MCI: Mild cognitive impairment
NCD: Noncommunicable diseases
HRQoL: Health-related quality of life
LE: Life expectancy
HRS: Health and Retirement Study
ROC: Receiver operating characteristic
EQ-5D: EuroQol-5 Dimensions instrument
PSM: Propensity score matching
HRs: Hazard ratios
BMI: Body mass index (kg/m^2^)
ADL: Activities of daily living
IADL: Instrumental activities of daily living
CESD: Centre for Epidemiology Studies-Depression Scale
SroH: Self-report of health
SD: Standard deviation
