# Supplementary material for: Estimation of losses of quality-adjusted life expectancy attributed to the combination of cognitive impairment and multimorbidity among Chinese adults aged 45 years and older
Source: BMC Public Health. 2021 Jan 5;21:24. doi: 10.1186/s12889-020-10069-w (PMC7786915; doi:10.1186/s12889-020-10069-w)
Supplement: Supplementary file 1 — Additional file 1: Appendix Table 1. Characteristics among groups of Chinese people ≥45 years of age included in this study - CHARLS (2011). [file 12889_2020_10069_MOESM1_ESM.docx]

| Appendix Table 1 –Characteristics among groups of Chinese people ≥ 45 years of age included in this study - CHARLS (2011) | | | | | | | | | | | | | | | |
| --- | --- | --- | --- | --- | --- | --- | --- | --- | --- | --- | --- | --- | --- | --- | --- |
| Demographic and health risk characteristic | |  | With multimorbidity (n = 6,087) | | | | |  | Without multimorbidity (n = 6,213) | | | | |  | Missing  (cognitive data)  (n =1,550) (%)* |
|  |  |  | Low cognition  (n = 1,766) (%)* | |  | High cognition  (n = 4,321) (%)* | |  | Low cognition  (n = 1,490) (%)* | |  | High cognition  (n = 4,723) (%)* | |  |  |
|  | Age groups (y), n (%) |  |  | |  |  | |  |  | |  |  | |  |  |
|  | 45-64 |  | 1,009(57.13) | |  | 3,207(74.22) | |  | 977(65.57) | |  | 4,047(85.69) | |  | 971(62.65) |
|  | 65-84 |  | 641(36.30) | |  | 1,046(24.21) | |  | 432(28.99) | |  | 642(13.59) | |  | 463(29.87) |
|  | ≥ 85 |  | 116(6.57) | |  | 68(1.57) | |  | 81(5.44) | |  | 34(0.72) | |  | 116(7.48) |
|  | Women, n (%) |  | 1,235(69.93) | |  | 2,070(47.91) | |  | 967(64.90) | |  | 2,150(45.52) | |  | 937(60.45) |
|  | Married & partnered, n (%) |  | 1,386(78.48) | |  | 3,881(89.82) | |  | 1,221(81.95) | |  | 4,361(92.34) | |  | 1,232(79.48) |
|  | Education, n (%) |  |  |  |  |  |  |  |  |  |  |  |  |  |  |
|  | Less than lower secondary |  | 1,747(98.92) | |  | 3,626(83.92) | |  | 1,470(98.66) | |  | 3,825(80.99) | |  | 1,429(92.19) |
|  | Upper secondary & vocational training |  | 17(0.96) | |  | 564(13.05) | |  | 18(1.21) | |  | 732(18.50) | |  | 97(6.26) |
|  | tertiary |  | 2(0.11) | |  | 131(3.03) | |  | 2(0.13) | |  | 165(3.49) | |  | 24(1.55) |
|  | Missing |  | 0(0.00) | |  | 0(0.00) | |  | 0(0.00) | |  | 1(0.02) | |  | 0(0.00) |
|  | Residence, n (%) |  |  |  |  |  |  |  |  |  |  |  |  |  |  |
|  | Rural Village |  | 1,564(88.56) | |  | 2,992(69.24) | |  | 1,379(92.55) | |  | 3,461(73.28) | |  | 1,320(85.16) |
|  | Urban Community |  | 201(11.38) | |  | 1,327(30.71) | |  | 111(7.45) | |  | 1,262(26.72) | |  | 230(14.84) |
|  | Missing |  | 1(0.06) | |  | 2(0.05) | |  | 0(0.00) | |  | 0(0.00) | |  | 0(0.00) |
|  | Smoking, n (%) |  |  |  |  |  |  |  |  |  |  |  |  |  |  |
|  | Never smoking |  | 1,223(69.25) | |  | 2,488(57.58) | |  | 1,019(68.39) | |  | 2,705(57.27) | |  | 1,040(67.10) |
|  | Smoked ever & Smoking |  | 543(30.75) | |  | 1,833(42.42) | |  | 470(31.54) | |  | 2,018(42.73) | |  | 510(32.90) |
|  | Missing |  | 0(0.00) | |  | 0(0.00) | |  | 1(0.07) | |  | 0(0.00) | |  | 0(0.00) |
|  | Drinking, n (%) |  |  |  |  |  |  |  |  |  |  |  |  |  |  |
|  | Never drinking |  | 1,380(78.14) | |  | 2,969(68.71) | |  | 1,087(72.95) | |  | 3,043(64.43) | |  | 1,179(76.06) |
|  | Drinked ever & Drinking |  | 386(21.86) | |  | 1,352(31.29) | |  | 403(27.05) | |  | 1,680(35.57) | |  | 371(23.94) |
|  | BMI, n (%) |  |  |  |  |  |  |  |  |  |  |  |  |  |  |
|  |  <18.5 |  | 129(7.30) | |  | 199(4.61) | |  | 135(9.06) | |  | 180(3.81) | |  | 140(9.03) |
|  | 18.5-23.9 |  | 776(43.94) | |  | 1,689(39.09) | |  | 808(54.23) | |  | 2,228(47.17) | |  | 625(40.32) |
|  | 24.0-26.9 |  | 321(18.18) | |  | 996(23.05) | |  | 232(15.57) | |  | 951(20.14) | |  | 252(16.26) |
|  | 27.0 |  | 294(16.65) | |  | 838(19.39) | |  | 130(8.72) | |  | 529(11.20) | |  | 152(9.81) |
|  | Missing |  | 246(13.93) | |  | 599(13.86) | |  | 185(12.42) | |  | 835(17.68) | |  | 381(24.58) |
|  | CHARLS (2011), baseline data of the China Health and Retirement Longitudinal Study;  * Percentages accounting for sampling proportion in subgroups  BMI, body mass index (kg/m^2^) ; | | | | | | | | | | | | | | |
