# Supplementary material for: Estimation of losses of quality-adjusted life expectancy attributed to the combination of cognitive impairment and multimorbidity among Chinese adults aged 45 years and older
Source: BMC Public Health. 2021 Jan 5;21:24. doi: 10.1186/s12889-020-10069-w (PMC7786915; doi:10.1186/s12889-020-10069-w)
Supplement: Supplementary file 2 — Additional file 2: Appendix Table 2. Losses of QALE at the individual level (with the corresponding 95% confidence intervals). [file 12889_2020_10069_MOESM2_ESM.docx]

Appendix Table 2 – Losses of QALE at the individual level (with the corresponding 95% confidence intervals)

| Age intervals  (y) |  | Losses of QALE at the Individual level | | | | | | | |
| --- | --- | --- | --- | --- | --- | --- | --- | --- | --- |
|  |  | Cognitive impairment  (n = 3,256) (95% CI) | |  | Multimorbidity ( n = 6,087) (95% CI) | |  | Cognitive impairment & Multimorbidity (n = 1,766) (95% CI) | |
| 45-49 |  | 3.10 | (2.29, 3.95) |  | 3.53 | (2.53, 4.56) |  | 7.61 | (5.68, 9.57) |
| 50-54 |  | 2.96 | (2.25, 3.70) |  | 3.27 | (2.38, 4.19) |  | 7.14 | (5.48, 8.83) |
| 55-59 |  | 2.83 | (2.22, 3.47) |  | 2.99 | (2.26, 3.74) |  | 6.75 | (5.32, 8.20) |
| 60-64 |  | 2.65 | (2.19, 3.13) |  | 2.83 | (2.12, 3.57) |  | 6.38 | (5.13, 7.65) |
| 65-69 |  | 2.44 | (2.17, 2.74) |  | 2.50 | (1.90, 3.13) |  | 5.83 | (4.88, 6.80) |
| 70-74 |  | 2.26 | (1.97, 2.57) |  | 2.31 | (1.84, 2.79) |  | 5.52 | (4.69, 6.35) |
| 75-79 |  | 1.95 | (1.73, 2.18) |  | 2.36 | (1.82, 2.91) |  | 5.40 | (4.58, 6.23) |
| 80-84 |  | 1.70 | (1.53, 1.88) |  | 2.24 | (1.92, 2.57) |  | 5.08 | (4.56, 5.62) |
| 85+ |  | 1.65 | (1.41, 1.89) |  | 1.80 | (1.67, 1.93) |  | 4.23 | (3.96, 4.51) |
| CI, confidence interval;  QALE, quality-adjusted life expectancy;  These losses are displayed for cognitive impairment, multimorbidity, and the combination of cognitive impairment and multimorbidity. | | | | | | | | | |
