# Supplementary material for: Estimation of losses of quality-adjusted life expectancy attributed to the combination of cognitive impairment and multimorbidity among Chinese adults aged 45 years and older
Source: BMC Public Health. 2021 Jan 5;21:24. doi: 10.1186/s12889-020-10069-w (PMC7786915; doi:10.1186/s12889-020-10069-w)
Supplement: Supplementary file 3 — Additional file 3: Appendix Table 3. Losses of QALE at the population level (with the corresponding 95% confidence intervals). [file 12889_2020_10069_MOESM3_ESM.docx]

Appendix Table 3 –Losses of QALE at the population level (with the corresponding 95% confidence intervals)

| Age intervals  (y) |  | Losses of QALE at the Population level | | | | | | | |
| --- | --- | --- | --- | --- | --- | --- | --- | --- | --- |
|  |  | Cognitive impairment (n = 3,256) (95% CI) | |  | Multimorbidity ( n = 6,087) (95% CI) | |  | Cognitive impairment & Multimorbidity (n = 1,766) (95% CI) | |
| 45-49 |  | 1.71 | (1.32, 2.13) |  | 1.91 | (1.24, 2.63) |  | 4.30 | (3.43, 5.20) |
| 50-54 |  | 1.71 | (1.33, 2.12) |  | 1.83 | (1.19, 2.51) |  | 4.25 | (3.41, 5.11) |
| 55-59 |  | 1.69 | (1.33, 2.08) |  | 1.70 | (1.12, 2.32) |  | 4.16 | (3.37, 4.91) |
| 60-64 |  | 1.65 | (1.34, 1.98) |  | 1.62 | (1.06, 2.20) |  | 4.04 | (3.34, 4.76) |
| 65-69 |  | 1.64 | (1.37, 1.93) |  | 1.46 | (0.96, 1.98) |  | 3.95 | (3.34, 4.59) |
| 70-74 |  | 1.63 | (1.34, 1.92) |  | 1.36 | (0.94, 1.79) |  | 3.88 | (3.33, 4.45) |
| 75-79 |  | 1.53 | (1.29, 1.79) |  | 1.42 | (0.97, 1.88) |  | 3.94 | (3.35, 4.54) |
| 80-84 |  | 1.45 | (1.22, 1.69) |  | 1.36 | (1.05, 1.68) |  | 3.86 | (3.40, 4.33) |
| 85+ |  | 1.45 | (1.17, 1.73) |  | 1.20 | (1.02, 1.37) |  | 3.46 | (3.20, 3.72) |
| CI, confidence interval;  QALE, quality-adjusted life expectancy | | | | | | | | | |

These losses displayed for cognitive impairment, multimorbidity, and the combination of cognitive impairment and multimorbidity.
