# Supplementary material for: Estimation of losses of quality-adjusted life expectancy attributed to the combination of cognitive impairment and multimorbidity among Chinese adults aged 45 years and older
Source: BMC Public Health. 2021 Jan 5;21:24. doi: 10.1186/s12889-020-10069-w (PMC7786915; doi:10.1186/s12889-020-10069-w)
Supplement: Supplementary file 6 — Additional file 6: Appendix Table 6. Losses of QALE (all participants without cognitive information were classified into the high cognition level) at the individual level based on the sensitivity analysis (with the corresponding 95% confidence intervals). [file 12889_2020_10069_MOESM6_ESM.docx]

Appendix Table 6 – Losses of QALE at the individual level based on the sensitivity analysis (with the corresponding 95% confidence intervals)

| Age intervals  (y) |  | Losses of QALE at the Individual level | | | | | | | |
| --- | --- | --- | --- | --- | --- | --- | --- | --- | --- |
|  |  | Cognitive impairment  (n = 3,256) (95% CI) | |  | Multimorbidity ( n = 6,861) (95% CI) | |  | Cognitive impairment & Multimorbidity (n = 1,766) (95% CI) | |
| 45-49 |  | 2.60 | (2.11, 3.13) |  | 3.53 | (2.39, 4.70) |  | 7.12 | (5.53, 8.75) |
| 50-54 |  | 2.46 | (2.07, 2.88) |  | 3.27 | (2.26, 4.31) |  | 6.64 | (5.27,8.04) |
| 55-59 |  | 2.33 | (2.04, 2.64) |  | 2.99 | (2.14, 3.86) |  | 6.24 | (5.11, 7.40) |
| 60-64 |  | 2.18 | (2.01, 2.37) |  | 2.83 | (2.01, 3. 68) |  | 5.90 | (4.95, 6.88) |
| 65-69 |  | 1.97 | (1.98, 1.97) |  | 2.50 | (1.80, 3.22) |  | 5.33 | (4.66, 6.03) |
| 70-74 |  | 1.77 | (1.77, 1.78) |  | 2.31 | (1.76, 2.87) |  | 4.98 | (4.53, 5.45) |
| 75-79 |  | 1.47 | (1.42, 1.53) |  | 2.36 | (1.79, 2.94) |  | 4.80 | (4.41, 5.21) |
| 80-84 |  | 1.20 | (1.09, 1.32) |  | 2.24 | (1.91, 2.58) |  | 4.42 | (4.27, 4.58) |
| 85+ |  | 1.11 | (1.03, 1.19) |  | 1.80 | (1.65, 1.95) |  | 3.69 | (3.51, 3.87) |
| CI, confidence interval;  QALE, quality-adjusted life expectancy;  These losses are displayed for cognitive impairment, multimorbidity, and the combination of cognitive impairment and multimorbidity.  In this sensitivity analysis, we classified all participants without cognitive information into the high cognition level. | | | | | | | | | |
